# HIV-1 epitopes presented by MHC class I types associated with superior immune containment of viremia have highly constrained fitness landscapes

**DOI:** 10.1371/journal.ppat.1006541

**Published:** 2017-08-07

**Authors:** Aleksandr M. Gorin, Yushen Du, Franklin Y. Liu, Tian-Hao Zhang, Hwee L. Ng, Christian Hofmann, William G. Cumberland, Ren Sun, Otto O. Yang

**Affiliations:** 1 Department of Microbiology, Immunology & Molecular Genetics, David Geffen School of Medicine, University of California Los Angeles, Los Angeles, California, United States of America; 2 Department of Molecular and Medical Pharmacology, David Geffen School of Medicine, University of California Los Angeles, Los Angeles, California, United States of America; 3 Molecular Biology Institute, University of California Los Angeles, Los Angeles, California, United States of America; 4 Division of Infectious Diseases, David Geffen School of Medicine, University of California Los Angeles, Los Angeles, California, United States of America; 5 Department of Biostatistics, Fielding School of Public Health, University of California, Los Angeles, California, United States of America; 6 AIDS Healthcare Foundation, Los Angeles, California, United States of America; Vaccine Research Center, UNITED STATES

## Abstract

Certain Major Histocompatibility-I (MHC-I) types are associated with superior immune containment of HIV-1 infection by CD8^+^ cytotoxic T lymphocytes (CTLs), but the mechanisms mediating this containment are difficult to elucidate *in vivo*. Here we provide controlled assessments of fitness landscapes and CTL-imposed constraints for immunodominant epitopes presented by two protective (B*57 and B*27) and one non-protective (A*02) MHC-I types. Libraries of HIV-1 with saturation mutagenesis of CTL epitopes are propagated with and without CTL selective pressure to define the fitness landscapes for epitope mutation and escape from CTLs via deep sequencing. Immunodominant B*57- and B*27- present epitopes are highly limited in options for fit mutations, with most viable variants recognizable by CTLs, whereas an immunodominant A*02 epitope-presented is highly permissive for mutation, with many options for CTL evasion without loss of viability. Generally, options for evasion overlap considerably between CTL clones despite highly distinct T cell receptors. Finally, patterns of variant recognition suggest population-wide CTL selection for the A*02-presented epitope. Overall, these findings indicate that these protective MHC-I types yield CTL targeting of highly constrained epitopes, and underscore the importance of blocking public escape pathways for CTL-based interventions against HIV-1.

## Author summary

Certain MHC class I types are associated with superior immune containment of HIV-1, underscoring the importance of CD8^+^ cytotoxic T lymphocytes (CTLs). Epitope escape mutations for these types is limited, indicating reduced immune evasion. Two proposed mechanisms are: 1) CTL targeting of highly sequence-constrained epitopes, or 2) more promiscuous CTLs for epitope variation. However, the *in vivo* complexity of undefined starting virus, multiple targeted epitopes, polyclonal CTL responses against each epitope, and post-hoc evaluation of the interaction renders examination of mechanisms difficult. Here we approach this question with controlled prospective *in vitro* experiments using saturation mutagenesis of epitopes in clonal HIV-1, propagated in the absence or presence of CTL clones to define the options for epitope mutation and immune evasion by deep sequencing. We find that two immunodominant epitopes presented by protective MHC types are highly mutation-constrained compared to one presented by a non-protective MHC type, whereas CTL promiscuity for epitope variation is not appreciably different. These results suggest that these protective MHC types are associated with limited HIV-1 escape predominately due to intrinsic constraints on epitope mutation, and underscore the importance of focusing the CTL response on highly conserved epitopes for immunotherapies and vaccines.

## Introduction

HIV-1-specific CD8^+^ cytotoxic T-lymphocytes (CTLs) play a significant protective role in the pathogenesis of HIV-1 infection [1–3], but ultimately fail to prevent disease progression in most persons. Myriad failure mechanisms have been proposed, but given the remarkable mutation rate and sequence plasticity of HIV-1 [4,5], the major factor is viral epitope escape mutation resulting in a cascade of viral persistence, CTL exhaustion, dysfunction, and senescence in chronic infection [6]. Indeed, evasion of CTLs is the major determinant of viral evolution *in vivo* [7–10]. Moreover, the major histocompatibility complex class I (MHC-I) locus is the best defined genetic determinant of disease progression rate in genome-wide association [11–13] and epidemiologic studies [14,15], indicating that MHC-I-associated properties of CTLs are important determinants of their efficacy.

Several studies of persons with “protective” MHC-I types who contain viremia without treatment have shown limited variation in targeted epitopes. Some have suggested that these are limited escape mutations with high fitness costs, based on examination of a few epitope variants observed *in vivo* [16–24]. However, the generality and mechanisms behind this observation are unclear, and the contributions of viral versus immune constraints for HIV-1 escape from CTLs are incompletely understood. Properties of the targeted epitope could be important; HIV-1 sequence plasticity is not uniform and epitopes likely vary in their constraints for mutation [25]. Alternatively, properties of the CTLs could differ; it has been proposed that the T cell receptors (TCRs) associated with protective MHC-I types either have greater cross-reactivity for epitope mutants and thus better limit possibilities for escape [26–28], or rather are better matched to common epitope variants [29]. Thus it is unresolved whether the limited escape is due to properties of the epitopes versus CTLs.

Finally, CTL responses against a given epitope are generally comprised of multiple clones with differing TCRs [30,31]. Because individual clones recognizing the same epitope can vary in the recognition of different variants [32–34], it has been proposed that clonal breadth may be important for preventing escape [30], but protective MHC-I types do not appear to yield greater TCR breadth overall [31]. This suggests qualitative differences in the composition or function of TCRs, and it is unclear to what degree the constraints for HIV-1 to escape CTLs are shared (“public escape”) versus specific for each clone (“private escape”).

Such issues are difficult to address *in vivo*, where the CTL response is polyclonal, the starting sequences of HIV-1 are typically undefined, and it is impossible to normalize selective pressure between epitopes. Here we assess the effect of HIV-1-specific CTLs on the fitness landscape of viral epitope mutation at clonal resolution. Libraries of HIV-1 epitope mutants are propagated under selective pressure to define the options for immune escape for multiple CTL clones associated with protective and non-protective MHC-I types, addressing these issues with an experimentally controlled approach to reveal CTL escape pathways for HIV-1.

## Results

### Construction of HIV-1 epitope libraries

**Fig 1:**
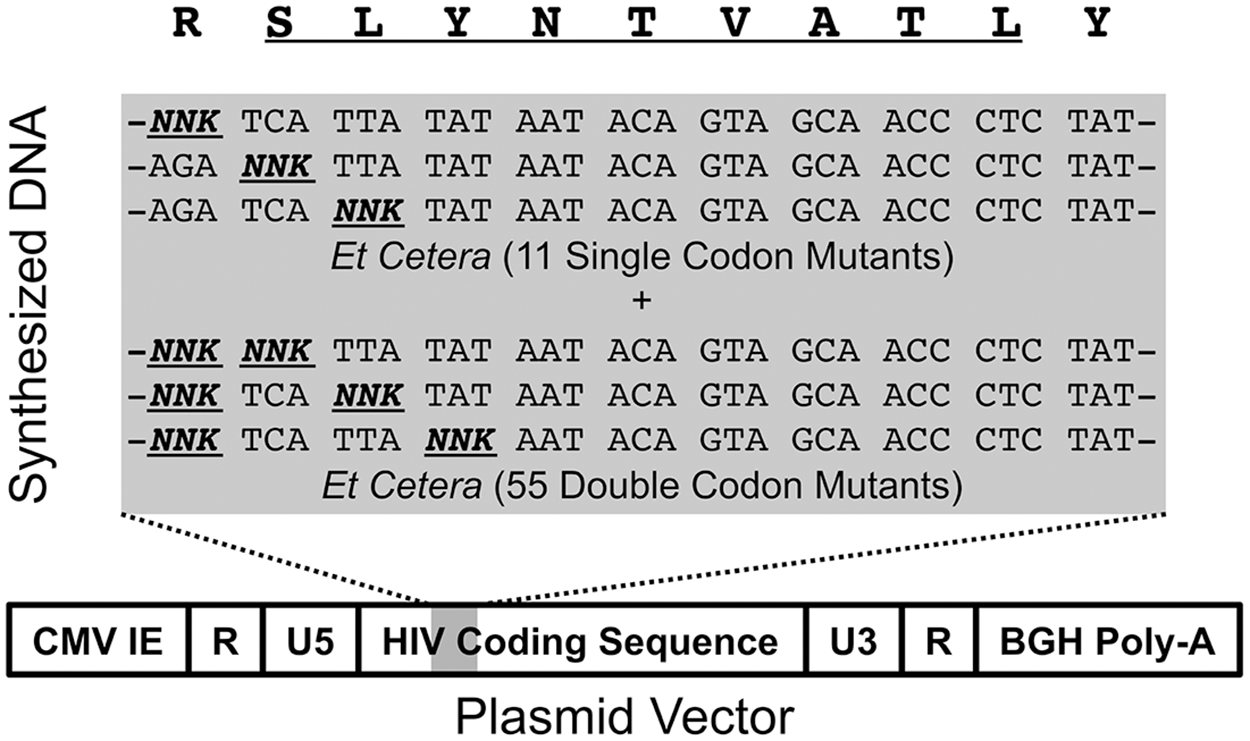
Production of HIV-1 epitope mutant virus libraries. Double-stranded DNA encoding an HIV-1 genomic region including an epitope of interest was synthesized to represent every possible single and double amino acid variant within the epitope and its immediately flanking amino acids, using degenerate codons (NNK, where N is any nucleotide and K is guanine or thymidine, encoding every amino acid while reducing stop codons). This DNA was then substituted into the full length HIV-1 NL4-3 genome to create a plasmid HIV-1 library for virus production.

**Fig 2:**
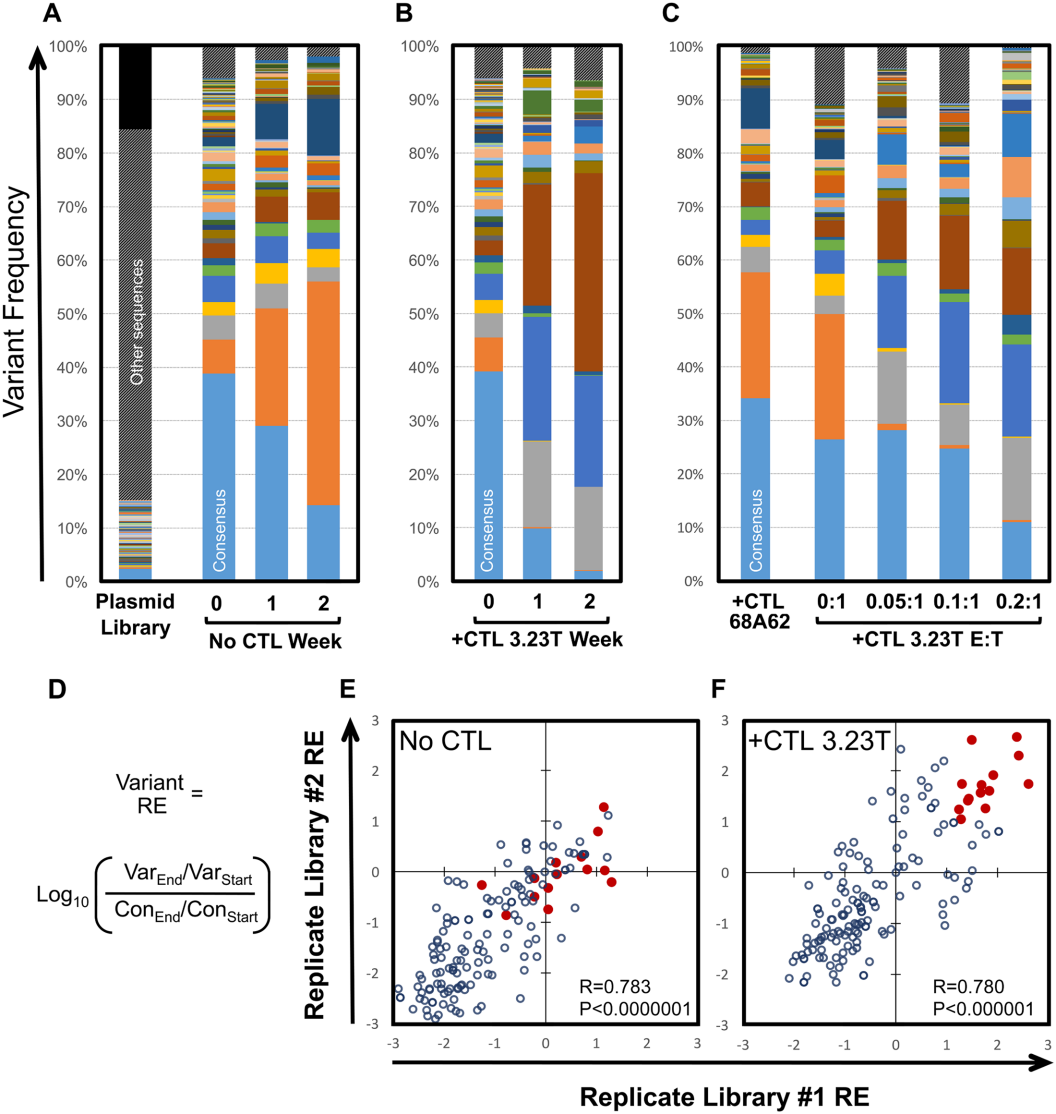
CTL selective pressure results in altered HIV-1 epitope variant frequencies. A. The frequencies of each SL9 epitope variant are plotted for the plasmid library, initial virus library, and virus populations after one or two weeks of passage (in the absence of CTL selection). All sequences below a frequency threshold of 2.5x10^-5^ in the plasmid library (inadequately represented for carry-through to the virus library) are represented in black. The frequencies of the consensus variant are significantly different between time points (p<0.00001). The subtype B consensus variant of the SL9 epitope is indicated by the bottom light blue bars in all bar graphs, and color coding of each variant is consistent across panels; variants not achieving the frequency cutoff of 1x10^-4^ in both replicates of the starting virus library are labeled “other sequences” and indicated by hatched gray bars. B. The frequencies of epitope variants after one and two weeks of passaging in the presence of the SL9-specific CTL clone 3.23T are indicated. Again, the consensus variant differs significantly between time points (p<0.00001), and additionally significantly different (p<0.00001) between libraries cultured without versus with 3.23T after 1 and 2 weeks. C. Epitope variant frequencies are plotted after one week of passaging in the presence of a control CTL clone 68A62 recognizing an A*02-restricted epitope in reverse transcriptase, or different effector to target (E:T) ratios of clone 3.23T. The frequencies of the consensus variant are significantly different (p<0.00001) between culture with 68A62 versus 3.23T. D. The formula to calculate relative enrichment (RE) of each variant compared to the subtype B consensus epitope variant using the frequency of each variant compared to the consensus epitope is shown. E and F. The REs between two biological replicate experiments with the SL9 variant library passaged in the absence (E) or presence (F) of clone 3.23T (RE_-CTL_ values without CTLs and RE_+CTL_ values with CTLs respectively) are plotted. Red dots in both panels E and F indicate variants with high RE_+CTL_ values, demonstrating their locations in the RE_-CTL_ plot. All panels are representative of individual replicate experiments.

Saturation mutagenesis was applied to three immunodominant HIV-1 epitopes in Gag (Table 1): SLYNTVATL (SL9, Gag 77–85, A*02-restricted), KAFSPEVIPMF (KF11, Gag 162–172, B*57-restricted), and KRWIILGLNK (KK10, Gag 263–272, B*27-restricted). Degenerate nucleotide DNA synthesis was utilized for each codon encoding the epitope and its flanking amino acids, as well as every combination of two codons, followed by substitution into the whole proviral genome of HIV-1 strain NL4-3 (Fig 1). The resulting plasmid libraries were found by deep sequencing to contain a full representation (100% for each epitope) of single amino acid variants and partial representation of double amino acid variants (38 to 43%) achieving the threshold frequency of 2.5x10^-5^ that was considered adequate for detectable virus production after transfection (Table 2). As expected, the consensus epitope sequence was overrepresented in each library because consensus amino acids were included in every degenerate codon (Fig 2A). These proviral DNA libraries were transfected into producer cells to yield starting virus libraries after a week of expansion. Deep sequencing of viral RNA in these libraries again demonstrated that the consensus epitope variant was predominant, but also demonstrated that a minority of the adequately represented variants in the plasmid library persisted as replication-competent variants (Table 2 and Fig 2A), suggesting that most epitope mutations were deleterious (36.4 to 86.6% of single codon mutants, 99.12 to 99.97% of double codon mutants). Epitope variants with a threshold frequency <10^−4^ in two experimental replicates of virus libraries were considered to be nonviable, because they tended to decay if present in only one library, indicating insufficient replicative capacity.

**Table 1:** HIV-1 epitopes and CTL clones.

Epitope (Abbreviation)	Gag Amino Acids (HXB2)	MHC-I Restriction	CTL Clone Designation^a^	TRBV	CDR3 Sequence	TRBJ
**SLYNTVATL** **(SL9)**	77–85	A*0201	S00001-SL9-**3.23T**	11–2	CASSLEHEQYF	2–7
			S00031-SL9-**10.11T**	12–3*01	CASSWEISDGYTF	1–2*01
			S00036-SL9-**1.9**	5–1*01	CASSFDSEQYF	2–7*01
**KAFSPEVIPMF** **(KF11)**	162–172	B*5701	S00014-KF11-**10.6**	15*02	CATSGTEYGYTF	1–2*01
			S00094-KF11-**3.4**	5–8*01	CASSVGFGANVLTF	2–6*01
**KRWIILGLNK** **(KK10)**	263–272	B*2705	S00076-KK10-**10.37**	27*01	CASREGQGALEQYF	2–7*01
			S00048-KK10-**TCR5**	7–9*03	CASSFDAGEQFF	2–1*01

^a^The underlined portion of the clone designation is utilized as an abbreviation throughout this study.

**Table 2:** Epitope variants contained in plasmid and virus libraries.

Epitope	Codon Mutants	Possible	In Plasmid Library	In Virus Library	Replicating
**SL9**	Single	209	209	77	36.8%
	Double	19,855	8,505	75	0.88%
	Both	20,064	8,714	152	1.74%
**KF11**	Single	247	247	33	13.4%
	Double	28,158	10,754	18	0.17%
	Both	28,405	10,901	51	0.46%
**KK10**	Single	228	228	145	63.6%
	Double	23,826	9,557	3	0.03%
	Both	24,054	9,785	148	1.51%

The number of possible single and double amino acid variants within each library (excluding stop codons) is given, as well as the number of these variants detected above threshold in the plasmid library (≥ 2.5x10^-5^, assumed to be adequate for representation after transfection of producer cells and passaging in 5 x 10^6^ cells thus yielding 20x sampling), and the number that carried forward above threshold (≥ 10^−4^ in both biological replicates, assumed to have replicated after transfection) in the virus libraries. Epitope variants below this frequency tended to decay if present in only one library, and thus were considered nonviable. If a variant was present above threshold in the plasmid library but inadequately represented in the virus library produced by transfection of the plasmid library into producer cells, it was also considered to be nonviable.

### Selection of epitope variants by CTL clones

To investigate the properties of the epitope variants, each library was passaged in the absence and presence of CTLs (Table 1) that had been confirmed to have antiviral activity in virus suppression assays (S1 Fig). Epitope sequences were obtained by deep sequencing at baseline and after each of two serial passages of one week each (S2 Fig). Significant shifts in the frequencies of epitope variants within a library occurred in the absence of CTLs, reflecting replicative capacity differences between variants (Fig 2A). Library propagation with the addition of epitope-targeted CTLs yielded distinctly different profiles of epitope variants, indicating superimposed selective pressure by the CTLs (Fig 2B). Control CTLs targeting an irrelevant epitope did not induce a profile distinct from passaging without CTLs, and the magnitude of the epitope-specific CTL-induced change was dose-dependent (Fig 2C). The small minority of variants containing stop codons that achieved the detectable threshold in the initial virus libraries generally showed sharply decaying frequencies (S3 Fig), confirming the reflection of replicative capacity. The outcome for each epitope variant was quantified as a relative enrichment value (RE) compared to the subtype B consensus epitope sequence, calculated as the log_10_ transformed ratio of frequencies normalized to subtype B consensus variant (Fig 2D) in the absence or presence of CTLs (RE_-CTL_ and RE_+CTL_ respectively). Thus RE_-CTL_ values reflected intrinsic replicative capacity relative to the consensus variant, with values <0 and >0 indicating variants replicating less and more efficiently (relative to the consensus variant) respectively. RE_+CTL_ values reflected the impact of CTL selection relative to the consensus variant, independently of replicative capacity (e.g. a variant with RE_-CTL_<0 and RE_+CTL_>0 indicates that it replicates less efficiently and is less suppressed by CTL than consensus). The REs between experimental replicates were highly correlated (Fig 2E and 2F), demonstrating the robustness of this measurement. Two separately produced virus libraries were utilized for all further determinations of RE_-CTL_ and RE_+CTL_ values, which were calculated as averages of quadruplicates (duplicate virus libraries each passaged in duplicate without CTLs) and duplicates (duplicate virus libraries each passaged singly with CTLs) respectively.

### Consistent patterns of epitope selection are seen in the absence and presence of CTL selective pressure

**Fig 3:**
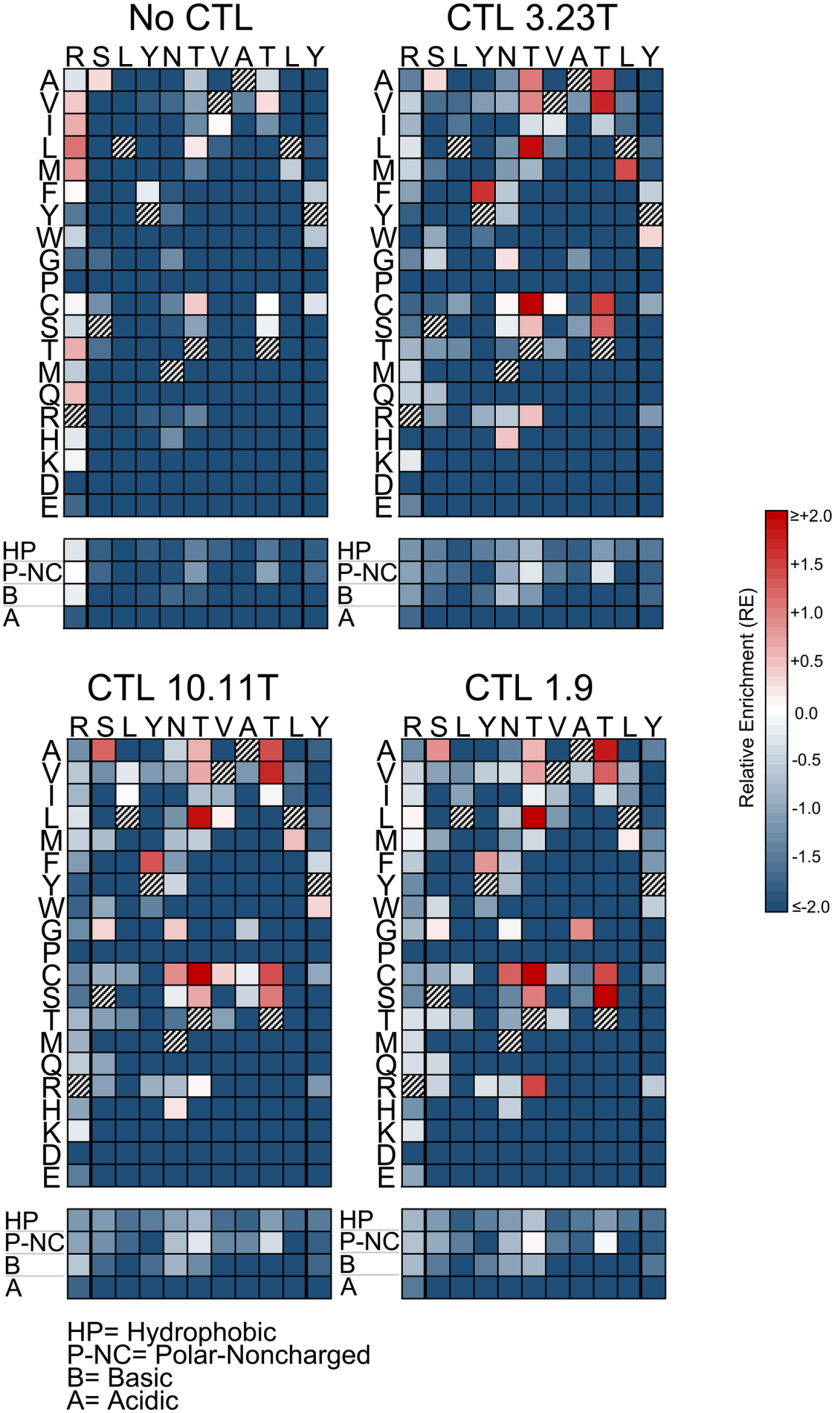
Single amino acid epitope variants display distinct patterns of selection in the absence or presence of CTLs (SL9 epitope). The RE values of all single amino acid variants with or without addition of the indicated CTL clones are displayed as color-scaled boxes. The horizontal axis indicates each subtype B consensus amino acid of each epitope and its immediately flanking amino acids, and the vertical axis indicates substituting amino acids. Hatched boxes indicate consensus amino acids. Additionally, the mean REs for substitutions of amino acids that are hydrophobic (A, V, I, L, M, F, Y, W, G, and P), polar-noncharged (C, S, T, N, and Q), basic (R, H, K), or acidic (D and E) are indicated below each plot. Variants that were detected above threshold in the plasmid library but not in the virus library were considered non-replicating and assigned RE_-CTL_ = -2.0 for these analyses.

**Fig 4:**
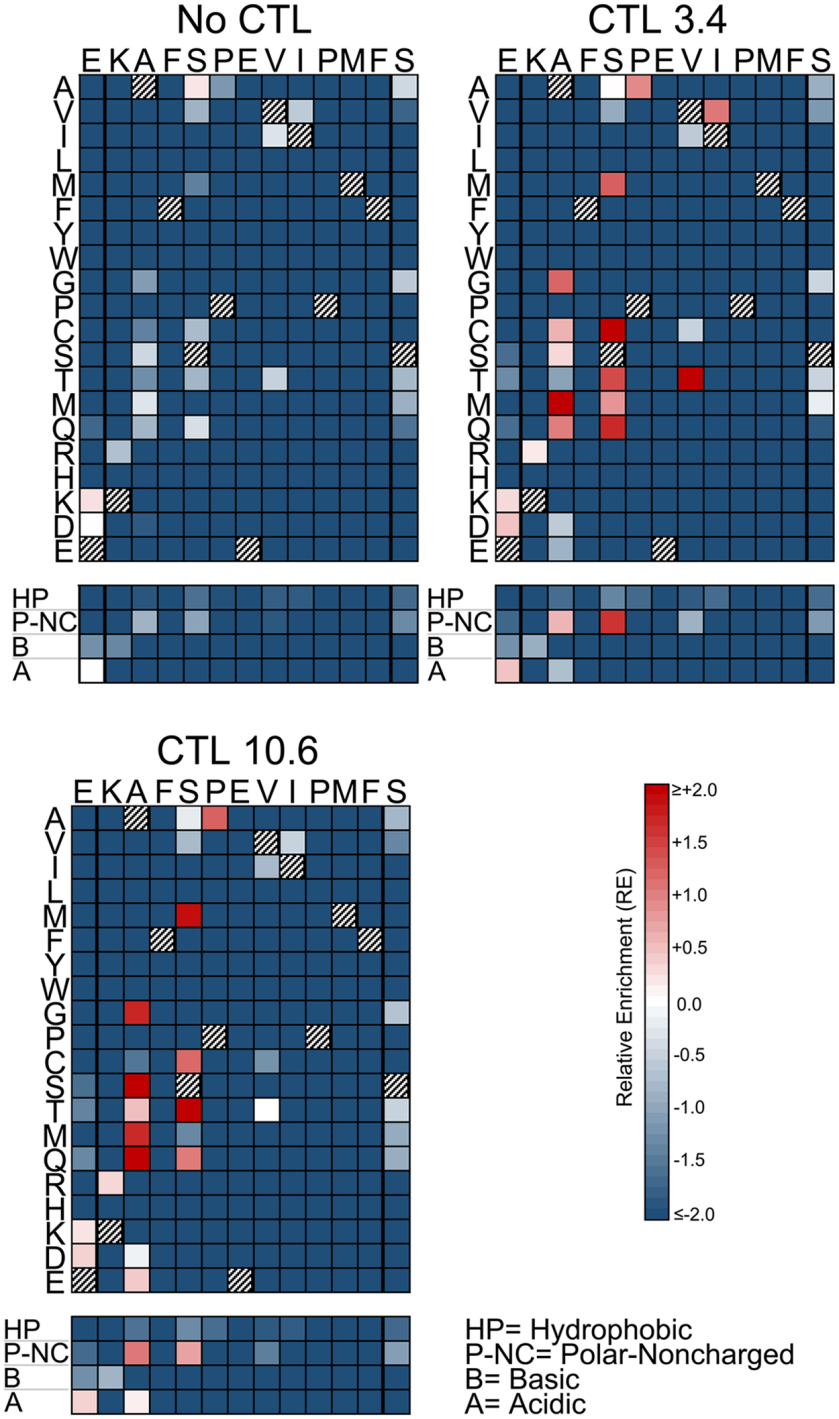
Single amino acid epitope variants display distinct patterns of selection in the absence or presence of CTLs (KF11 epitope). The RE values of all single amino acid variants with or without addition of the indicated CTL clones are displayed as color-scaled boxes. The horizontal axis indicates each subtype B consensus amino acid of each epitope and its immediately flanking amino acids, and the vertical axis indicates substituting amino acids. Hatched boxes indicate consensus amino acids. Additionally, the mean REs for substitutions of amino acids that are hydrophobic (A, V, I, L, M, F, Y, W, G, and P), polar-noncharged (C, S, T, N, and Q), basic (R, H, K), or acidic (D and E) are indicated below each plot. Variants that were detected above threshold in the plasmid library but not in the virus library were considered non-replicating and assigned RE_-CTL_ = -2.0 for these analyses.

**Fig 5:**
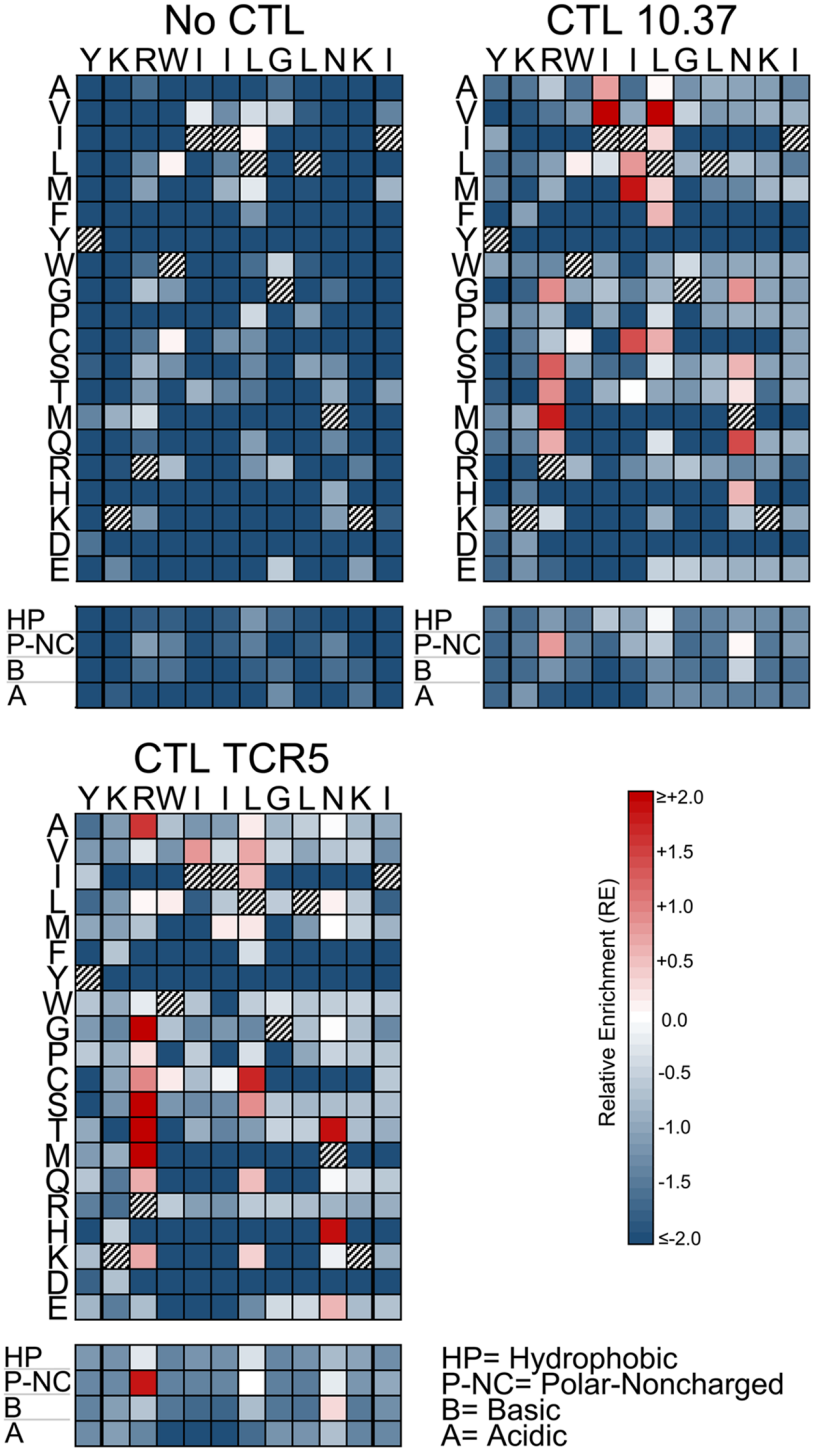
Single amino acid epitope variants display distinct patterns of selection in the absence or presence of CTLs (KK10 epitope). The RE values of all single amino acid variants with or without addition of the indicated CTL clones are displayed as color-scaled boxes. The horizontal axis indicates each subtype B consensus amino acid of each epitope and its immediately flanking amino acids, and the vertical axis indicates substituting amino acids. Hatched boxes indicate consensus amino acids. Additionally, the mean REs for substitutions of amino acids that are hydrophobic (A, V, I, L, M, F, Y, W, G, and P), polar-noncharged (C, S, T, N, and Q), basic (R, H, K), or acidic (D and E) are indicated below each plot. Variants that were detected above threshold in the plasmid library but not in the virus library were considered non-replicating and assigned RE_-CTL_ = -2.0 for these analyses.

**Fig 6:**
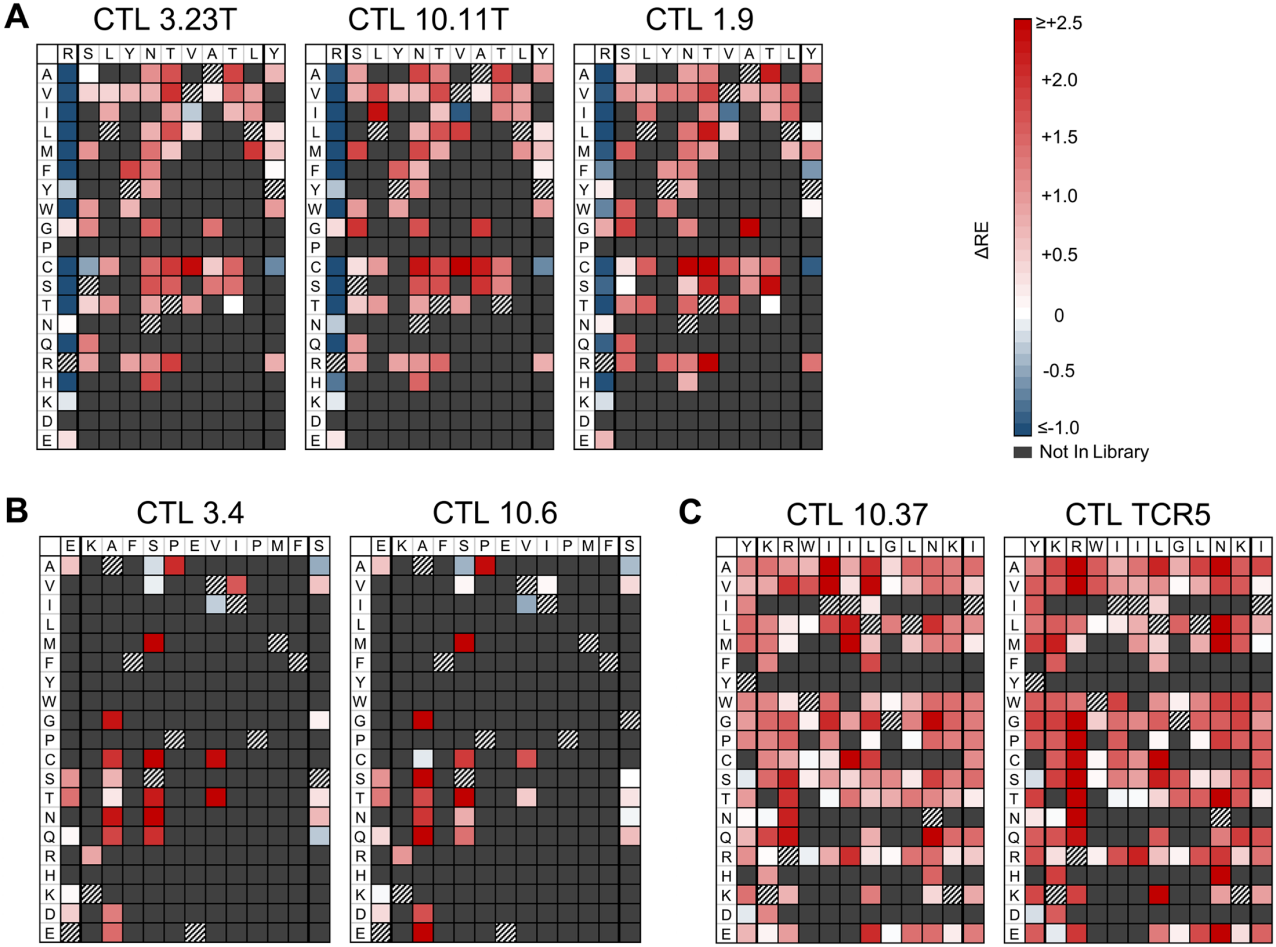
Identification of single amino acid epitope variants that potentially escape from CTLs. The net changes in RE values due to CTL selection (ΔRE = RE_+CTL_—RE_-CTL_) are displayed as color-scaled boxes for all single amino acid variants of epitopes SL9 (A), KF11 (B), and KK10 (C). The horizontal axis indicates each subtype B consensus amino acid and its immediately flanking amino acids, and the vertical axis indicates substituting amino acids. Variants that detected above threshold in the plasmid library but not in the virus library are considered non-replicating and coded by dark grey. Consensus amino acids are coded by hatched boxes.

**Fig 7:**
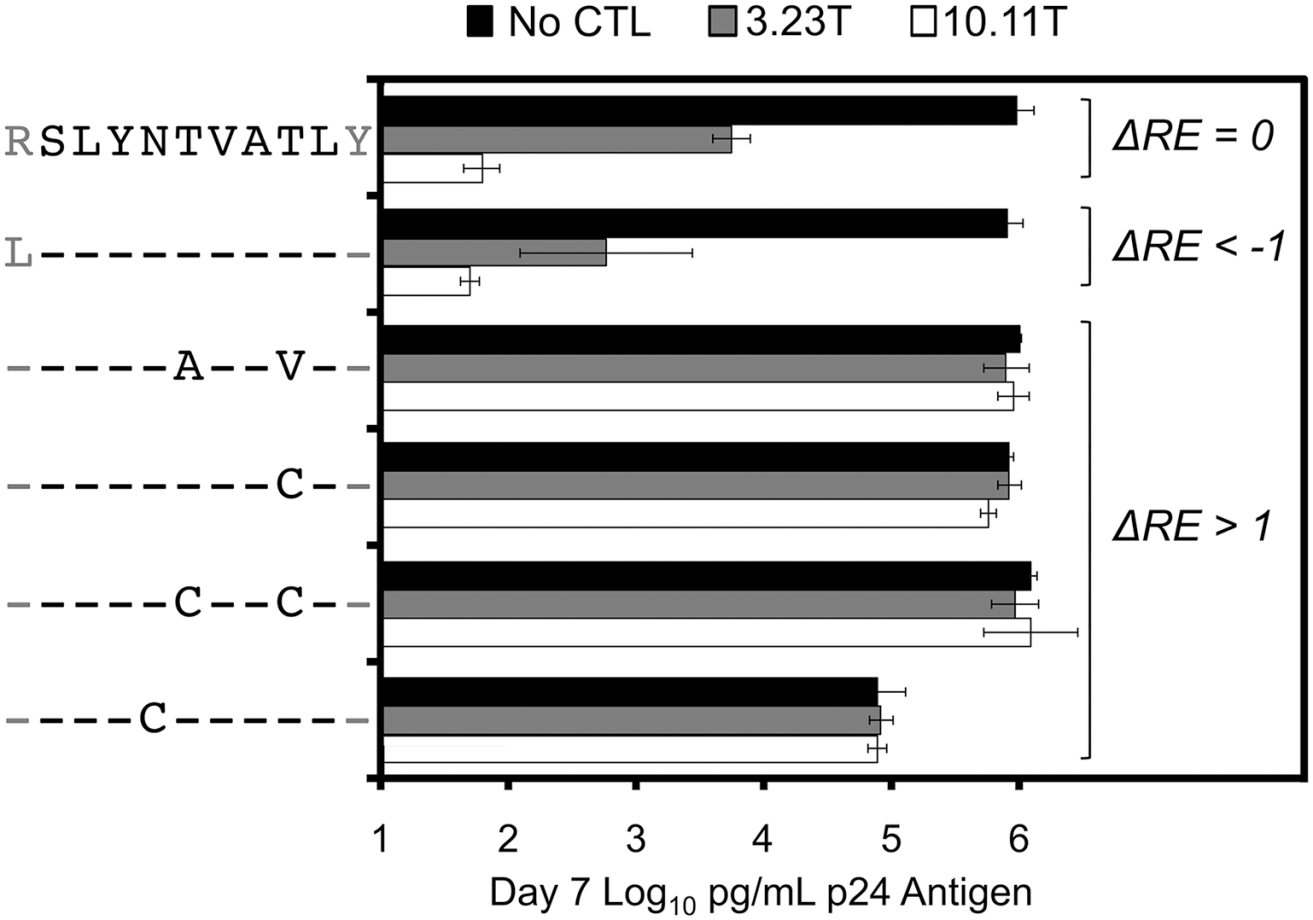
Confirmation that ΔRE values reflect potential for escape from CTLs. Clonal HIV-1_NL4-3_ with five SL9 epitope variants with increased (1.39–1.66 and 1.37–2.32 for CTL clones 3.23T and 10.11T respectively), one variant with decreased (-1.42 and -1.43 with CTL clones 3.23T and 10.11T respectively) ΔRE, and the subtype B consensus epitope were individually tested for susceptibility for suppression by CTLs. T1 cells were acutely infected and cultured in the presence or absence of CTL clones 3.23T or 10.11T; supernatant p24 antigen values after 7 days are plotted for each indicated variant. Each value is the mean of triplicates, and error bars represent standard deviations.

The impacts of mutations at each epitope amino acid position were evaluated by examining the subsets of single codon mutants in each library (Figs 3–5). Passaging in the absence of CTLs revealed the effects of point mutations on intrinsic viral replication. For each epitope, most mutations had negative effects on replication (RE_-CTL_<0). However, each epitope also demonstrated mutations that were tolerated or advantageous (RE_-CTL_≥0). For SL9, substitutions at multiple positions yielded enrichment, particularly at residues -1, 5, and 8 of the epitope (Fig 3 “No CTL” panel). KF11 appeared to have fewer tolerated mutations (Fig 4 “No CTL” panel), mostly at residues 2 and 4, while KK10 (Fig 5 “No CTL” panel) had several tolerated mutations mostly at residues 2, 5, and 6. Evaluation of these epitopes under additional CTL selection also demonstrated patterns of epitope enrichment relative to the consensus epitope sequences (RE_+CTL_ >0). The addition of CTL generally appeared to augment enrichment of epitope variants with intrinsic growth advantages in the absence of CTLs (Figs 3–5), although there were also some intrinsically disadvantageous variants that gained enrichment with the addition of CTLs. Conversely, some intrinsically advantageous variants were selected against with the addition of CTLs, particularly those with substitutions at the -1 position of the SL9 epitope. The net effect of CTL selection (ΔRE = RE_+CTL_- RE_-CTL_) was examined for each epitope variant (Fig 6). The relevance of this value to identify potential CTL escape variants was confirmed by generating HIV-1 clones corresponding to library variants with defined ΔRE values, and testing their susceptibility to inhibition of replication by CTLs (Fig 7). Thus this parameter showed that many single substitutions conferred benefits against CTL selection (Fig 6). A major exception was the N-terminal flanking amino acid of the SL9 epitope (position -1), where most substitutions increased susceptibility to CTLs. Overall, these data demonstrate epitope-specific constraints for mutation and evasion of CTLs.

### KF11 and KK10 escape from CTLs is more constrained than SL9 mostly due to more limited fitness landscapes for epitope mutation

**Fig 8:**
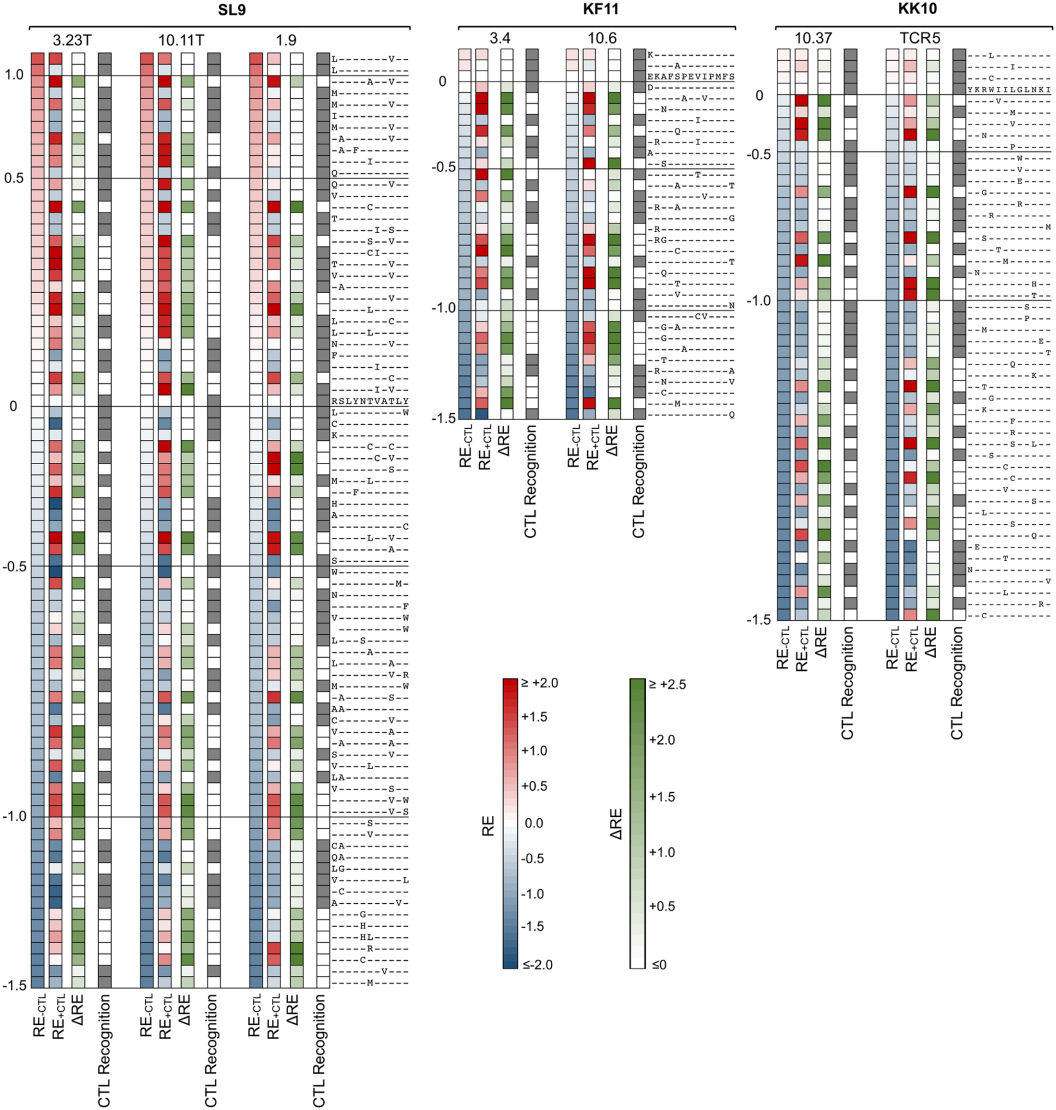
CTL selection of all library epitope variants. The RE values of all epitope variants with or without addition of the indicated CTL clones are displayed as color-scaled boxes for epitopes SL9, KF11, and KK10. The horizontal axis indicates each consensus amino acid and its immediately flanking amino acids, and the vertical axis indicates substituting amino acids. First columns indicate relative enrichment versus consensus variant without CTLs (RE_-CTL_), second columns indicate relative enrichment versus consensus variant with added CTLs (RE_+CTL_), third columns indicate the difference between those values (ΔRE = RE_+CTL_-RE_-CTL_), and fourth columns indicate variants with ΔRE ≥ 0.7 = 5-fold (white boxes) or < 0.7 (gray boxes). Variants with RE_-CTL_ < -1.5 and stop codons are omitted.

**Fig 9:**
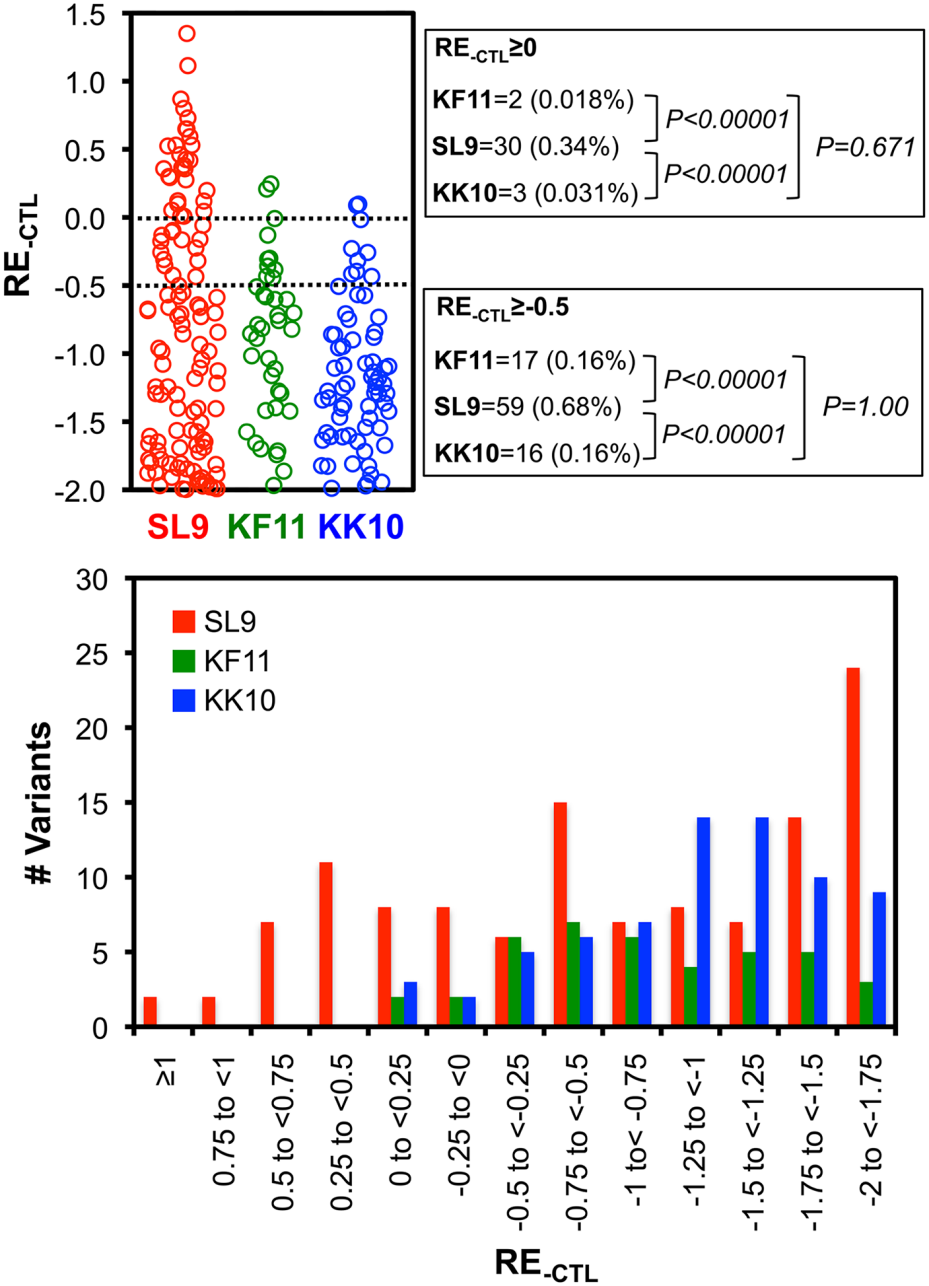
Distributions of epitope variant viabilities. Top panel: The mean RE_-CTL_ values of all variants in the virus library are plotted for each epitope. The numbers achieving thresholds of RE_-CTL_≥0 and RE_-CTL_≥-0.5 and their frequencies (over all variants adequately represented in the plasmid library) are indicated for each epitope. Variants with RE_-CTL_<-2 are not plotted. Bottom panel: The numbers of variants in different ranges of mean RE_-CTL_ values are indicated.

**Fig 10:**
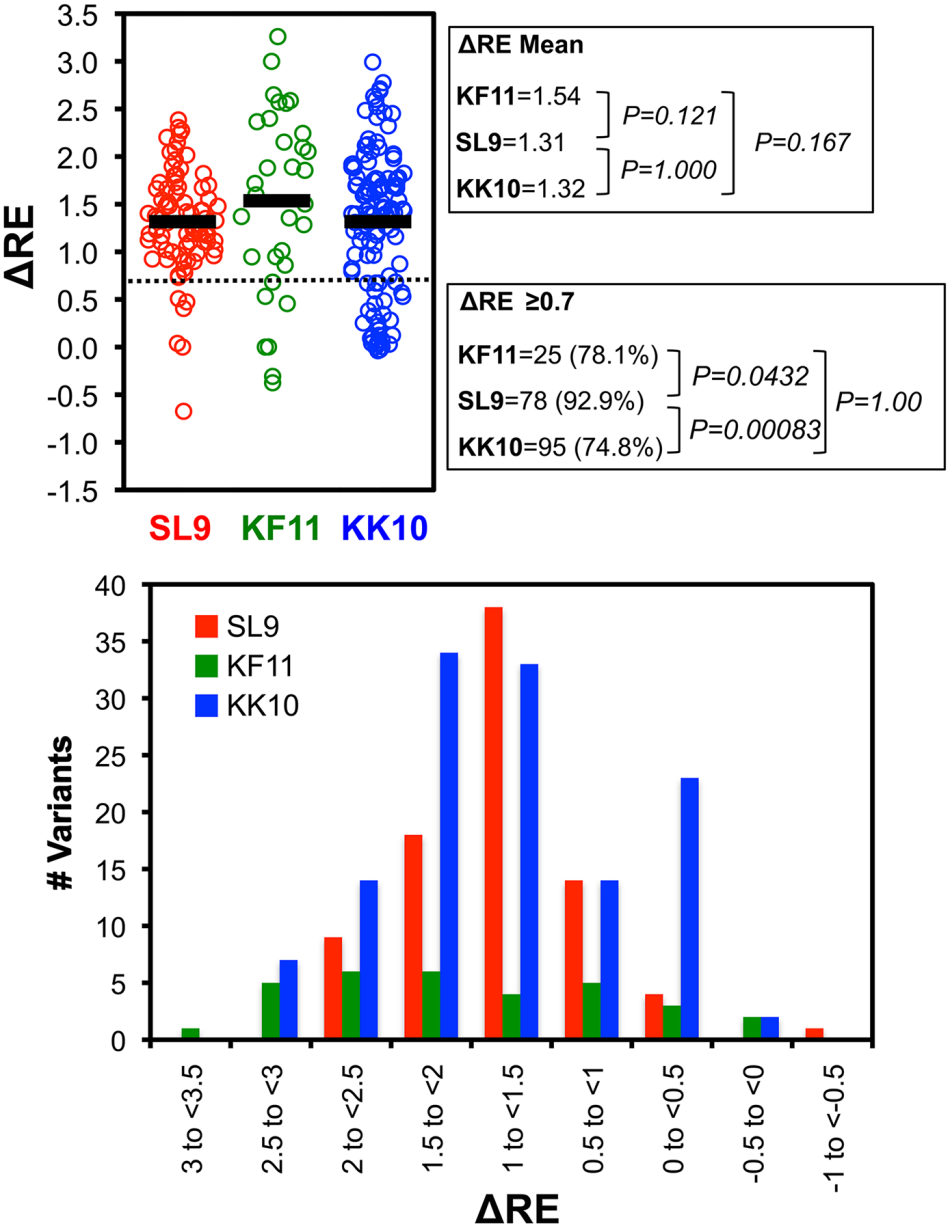
CTL promiscuity for epitope variant recognition. Top panel: The mean ΔRE values of all variants in the virus library are plotted for each epitope, excluding those with mutations in the epitope flanking residues. The numbers and frequencies (over all variants adequately represented in the virus library) of variants achieving a threshold of ΔRE≥0.7 are indicated. Bottom panel: The numbers of variants in different ranges of mean ΔRE values are indicated.

**Fig 11:**
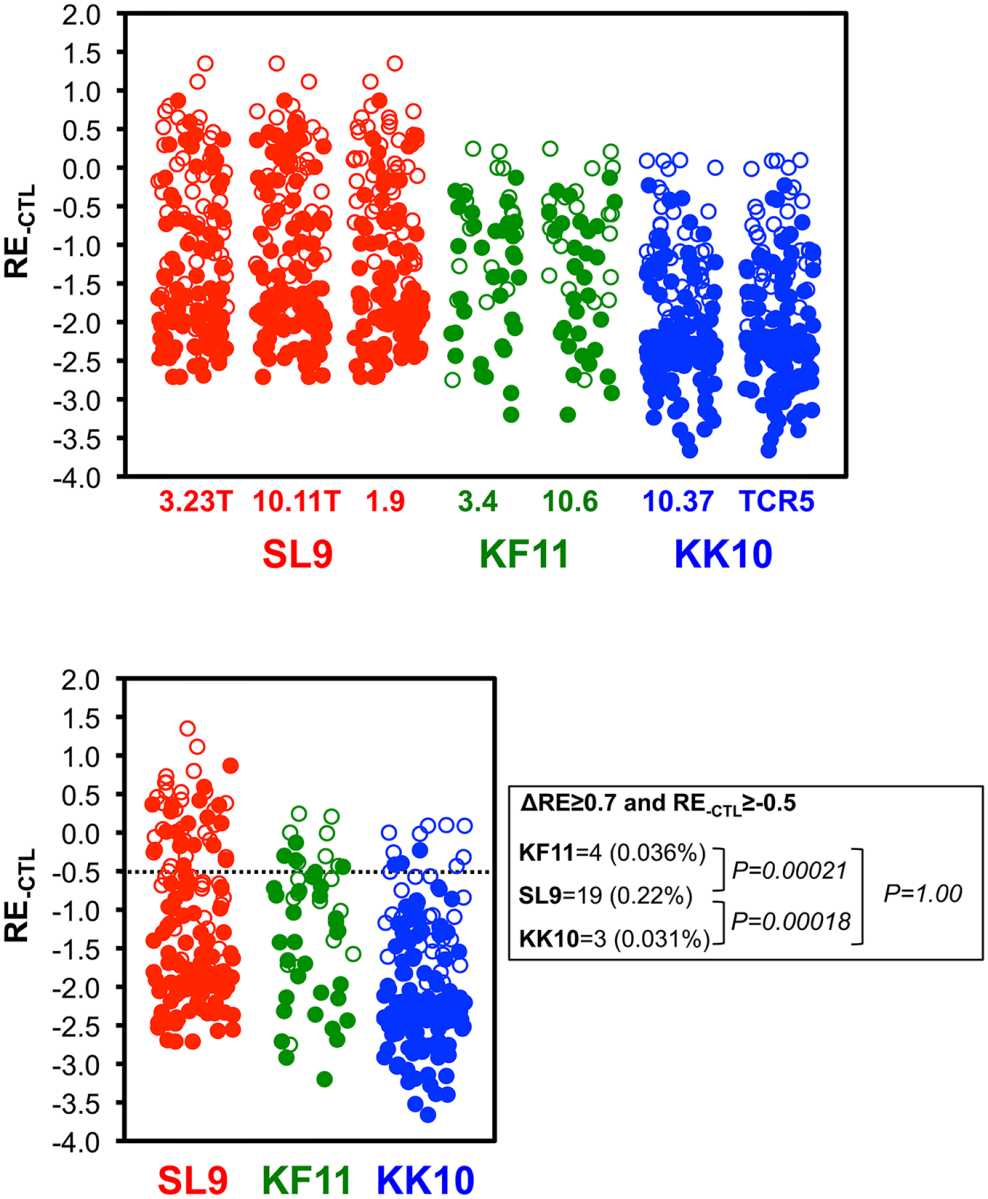
Comparisons of viabilities of potential escape variant epitopes for SL9, KF11, and KK10. Top panel: RE_-CTL_ values are plotted for all SL9, KF11, and KK10 epitope variants (excluding those with substitutions in flanking residues) in the virus libraries. Potential escape variants (ΔRE≥0.7) are indicated by filled symbols, and the remainder are open symbols. Bottom panel: RE_-CTL_ values are plotted for all SL9, KF11, and KK10 epitope variants as above, where variants with mean ΔRE≥0.7 for all tested CTLs are indicated by filled symbols. Numbers of potential escape variants with RE_-CTL_>-0.5 and their frequencies among variants in the virus libraries are indicated.

**Fig 12:**
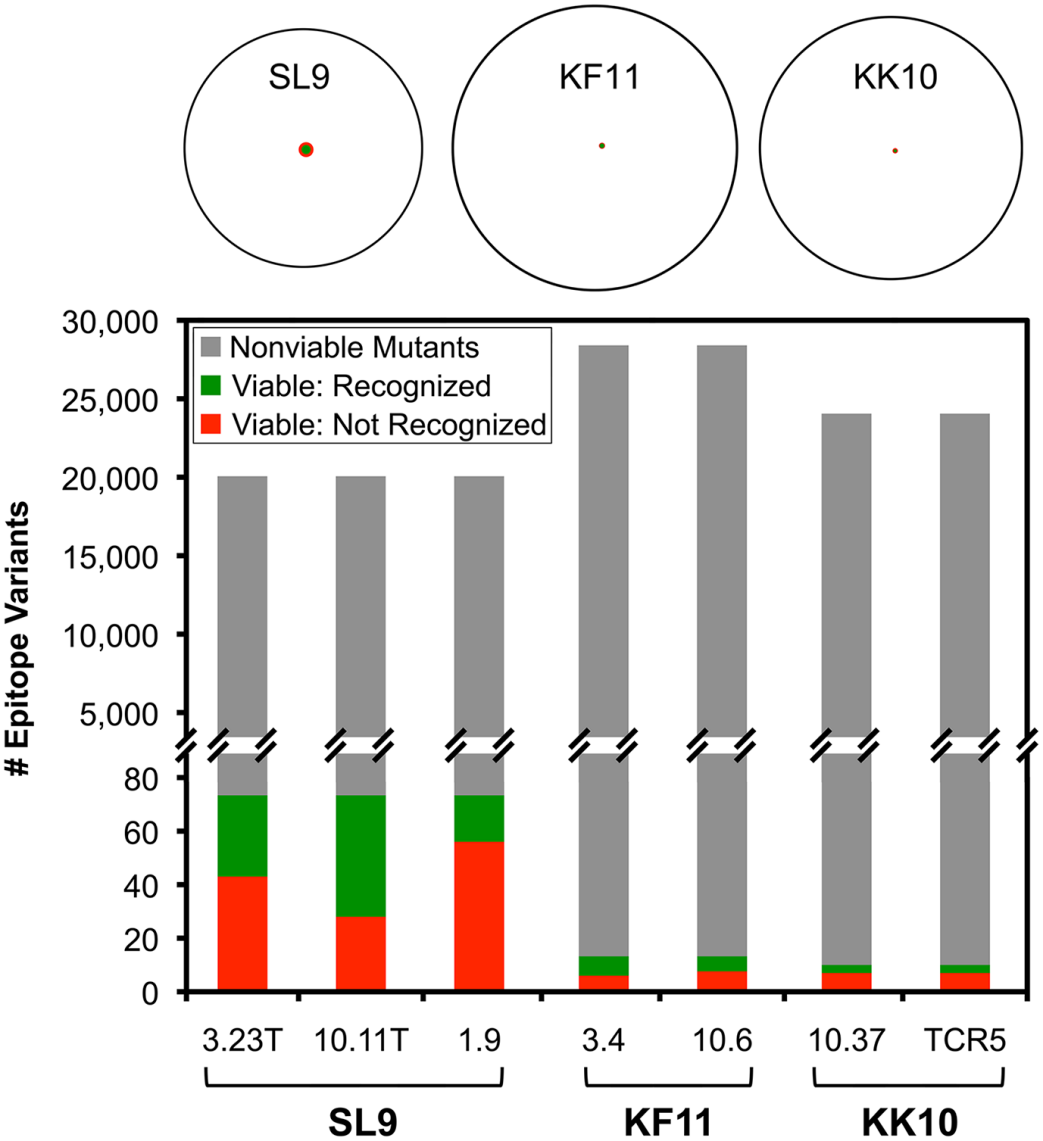
Schematic representation of fitness landscapes and CTL escape options for SL9, KF11, and KK10. Epitope variants (including the immediate flanking residues) with mean ΔRE≥0.7 were considered potential escape variants, and RE_-CTL_≥-0.5 were considered viable. The percentages of viability and susceptibility to CTLs for double amino acid variants that were missing in our plasmid library were inferred to be the same as those that were present and tested in this study. Top row: Venn diagrams indicate total numbers of possible single and double amino acid variants (white circles), numbers of viable variants (red circles), and numbers of viable variants susceptible to CTLs (green circles). The surface areas of each circle are approximately proportional to the numbers of variants contained. Bottom panel: Bar graphs indicate the same data, where gray bars indicate non-viable variants, green bars indicate viable variants that are recognized by CTLs, and red bars indicate viable variants that escape CTLs.

Quantitative analyses were extended to all epitope variants in the libraries, including double amino acid mutants (Table 1), to compare epitopes. First examining RE_-CTL_ (Fig 8, S4–S6 Figs first columns), the SL9 library yielded more variants with neutral to moderately decreased replication capacity (RE_-CTL_≥0 or RE_-CTL_≥-0.5) compared to KF11 and KK10, whereas KF11 and KK10 were similar (Fig 9). There were 30 and 59 (0.34% and 0.68% of all single and double SL9 mutants adequately represented in the plasmid library) SL9 variants with RE_-CTL_ ≥0 and -0.5 respectively, compared to 2 and 17 (0.018% and 0.031%) and 3 and 16 (0.16% and 0.16%) of KF11 and KK10 epitopes reaching those thresholds (Fig 9 top). The distributions of measurements showed increasing numbers of lower RE_-CTL_ variants, consistent with insufficient replicative capacity for the variants in the plasmid library that were not detected in the virus library (Fig 9 bottom). Comparing susceptibilities of epitope variants to CTLs (ΔRE), many variants had neutral to enriched effects under CTL selection (Fig 8). Across all variants in the virus libraries (excluding variants with mutations in epitope flanking residues, to isolate effects of changes in CTL epitope recognition from epitope processing), this parameter displayed a range of values that was normally distributed (Fig 10). The mean ΔRE value across all SL9-specific CTLs was similar to KF11- and KK10- specific CTLs (1.31 versus 1.54 and 1.32 respectively), although the percentages of variants with at least 5-fold advantage under CTL selection (ΔRE≥0.7) was significantly higher (92.9% versus 78.1% and 74.8% respectively, Fig 10 top). Over the range of 2-to 10-fold relative enrichment with CTLs, a stable profile of selected variants was observed (S4-S6 third columns), and thus 5-fold selection (ΔRE≥0.7) was chosen as a definition of potential escape. Finally, considering the numbers of potential escape variants under this definition with at least moderate replicative capacity (RE_-CTL_≥-0.5) as viable options for escape, SL9 had significantly more options than KF11 or KK10. Using mean values across CTLs, SL9 had 19 variants (0.22% of variants in the virus library) compared to 4 (0.036%) and 3 (0.031%) variants for KF11 and KK10 respectively meeting these criteria (Fig 11). In summary, the SL9 epitope offers more options for viable mutations than KF11 or KK10, and average CTL coverage of those mutations is similar or perhaps modestly decreased for SL9 compared to KF11 and KK10 (Fig 12).

#### Different CTL clones targeting each epitope overlap considerably in escape mutations

**Fig 13:**
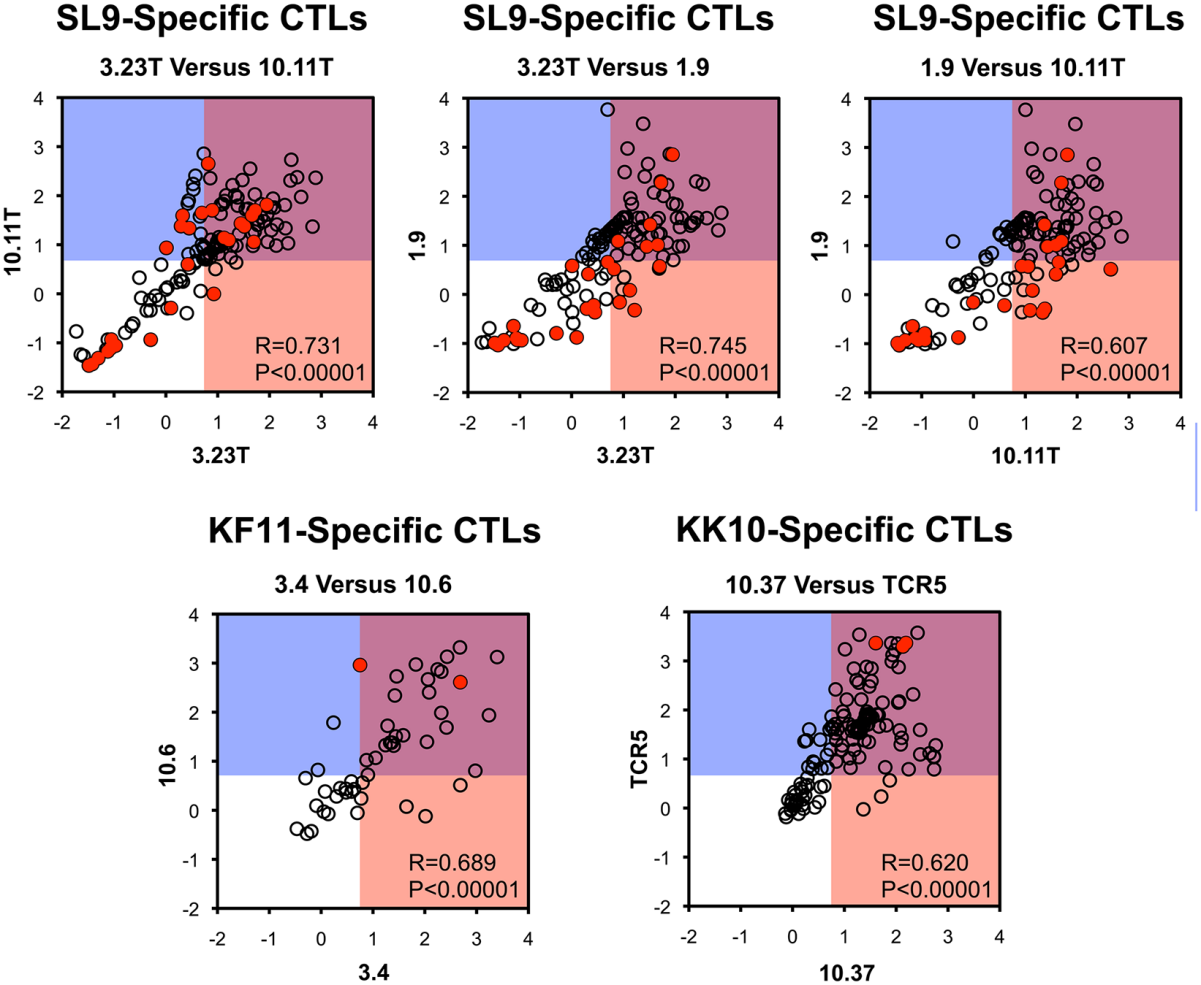
Correlations of epitope variant susceptibilities to different CTLs targeting SL9, KF11, or KK10 epitopes. The ΔRE values are compared between CTLs targeting targeting each epitope. Shaded areas indicate ΔRE≥0.7 for one or both CTLs, and red filled symbols indicate those with RE-CTL_≥-0.5_. Epitope variants that were not detected in the virus libraries (non-replicating) were excluded.

To address the similarity of epitope variant recognition between differing CTL clones, the ΔRE values for clones were compared (Fig 13). In general, CTL susceptibilities of variants were highly correlated for SL9, KF11, and KK10 epitopes, with few exceptions. Overall, these data suggest that most potential escape mutations for each epitope are shared across different CTL clones (representing “public” options).

## Discussion

This study addresses the fitness landscape for mutational variation of three HIV-1 epitopes and the restrictions imposed by CTLs. While *in vivo* observations have revealed the effects of CTL on viral evolution to escape, our data dissect this process in greater detail, resolving the interaction at the level of individual CTL clones and defined starting virus quasispecies populations. For each epitope, the effect of every single amino acid polymorphism (as well as about a third of all double amino acid polymorphisms) versus the subtype B consensus sequence is assessed by frequency change as a reflection of fitness during serial passaging, as well as the impact of clonal CTL selection on these variants. Two epitopes presented by protective MHC-I types B*57 (KF11) and B*27 (KK10) and an epitope presented by the non-protective type A*02 (SL9) are examined in detail.

The quantities of mutation options in the absence of CTL selection markedly differ between these epitopes. The SL9 epitope exhibits many variants with similar or higher fitness compared to consensus, whereas KF11 and KK10 epitopes appear to have very few. This finding indicates that the SL9 epitope is much less constrained for mutation than KF11 and KK10, suggesting that HIV-1 generally has fewer options for mutational escape in KF11 and KK10 (Gag p24) than SL9 (Gag p17) epitopes. This result agrees with prior observations that: efficient immune containment of HIV-1 corresponds to CTL targeting of p24 [35], that immunodominance of p24 targeting is commonly associated with protective MHC-I types (including B*57 and B*27) [36,37], and that p24 is highly conserved overall [25]. However, studies delineating associations of particular CTL responses with immune containment of HIV-1 demonstrate that protective epitope targeting is not limited to p24 [37,38], suggesting that sequence constraint at the level of the individual epitope overrides the particular source protein in importance for escape and thus CTL efficacy.

The epitope variants that were enriched under CTL selection further illuminate the constraints for escape mutation. For SL9, there are several highly CTL-enriched variants with intrinsic fitness near the consensus epitope. In contrast, KF11 and KK10 both exhibit few CTL-enriched variants with preserved fitness, in agreement with prior studies showing that CTL escape mutations for these epitopes require high fitness costs [16–19,21–24]. Moreover, the variants enriched by CTL selection recapitulate several previously reported escape variants *in vivo*, such as Y79F in SL9 [16] and A163G in KF11 [18], although some other reported escape variants such as KF11 A163G/S165N [18] were present in the initial plasmid library but appeared replication incompetent. As a whole, these data support the concept that protective MHC-I types such as B*27 and B*57 are beneficial through generating CTL responses against epitopes for which escape occurs only at a high fitness cost to HIV-1.

Regarding the alternative hypothesis that protective MHC-I types yield TCRs with greater promiscuity for epitope variation [26–28], our findings do not provide definitive evidence. While KF11- and KK10- specific CTLs do appear to recognize more variants on average than SL9-specific CTLs, the average impacts of CTLs on epitope variants do not vary significantly between epitopes. However, these measurements are limited to CTL interactions only with viable variants, and are thus not a comprehensive evaluation of promiscuity across all epitope variation. Within the subset of viable mutants, there is no clear difference in coverage by CTLs across the three epitopes, and the findings are consistent with a study suggesting that better immune containment of HIV-1 is mediated by CTL responses that are more focused on viable epitope variants despite recognizing fewer epitope variants overall [29].

An unexpected finding is that CTL recognition of SL9 is enhanced by various substitutions at the N-terminus flanking amino acid. This suggests that these substitutions increase epitope presentation compared to the consensus sequence. Although the influence of various mutations within the SL9 epitope reducing its proteasomal processing and presentation have been demonstrated [39], the impairment of processing associated with the N-terminus flanking residue in the consensus sequence has not been reported. Given the high prevalence of A*02 and the capacity of other MHC-I types such as B*40 to present the SL9 epitope, it is plausible that the consensus sequence represents escape adaptation across the human population. Also unexpected is the observation that several SL9 epitope variants had apparently higher fitness than the consensus sequence. Both these findings support the proposal that HIV-1 can accumulate escape mutations in the consensus sequence for circulating strains, as has been suggested specifically for SL9 [40] and more generally across the HIV-1 genome [7,8].

We previously reported the differential ability of CTL clones targeting the same epitope to cross-recognize escape variants [32–34]. Here we confirm such differences between clones, but find that the overall options for escape are strikingly similar even between TCRs with entirely different variable chains. For each epitope, the amino acid substitutions resulting in CTL evasion follow stereotypic patterns mostly sparing the main MHC-I anchor-binding residues. Although such substitutions could affect proteasomal processing, epitope stability, or MHC-I binding, this suggests shared mutational pathways for ablating binding of sequence-distinct TCRs, and that these “public escape” pathways may predominate for these epitopes, consistent with prior population-based studies of HIV-1 escape “footprints” *in vivo* [8,41].

Several caveats must be considered for the interpretation of our data. Our libraries provide complete coverage for single amino acid polymorphisms in the epitopes, but incomplete coverage for double amino acid polymorphisms, and no coverage for three or more changes. However, most reported escape mutations are single or double polymorphisms compared to consensus, and our data show sharply decreased viability for double mutants compared to single mutants, suggesting that very few triple mutants would be viable. The RE values for epitope variants are semiquantitative reflections of HIV-1 fitness, given the saturating conditions for viral growth that can exaggerate the competitive advantage of the most fit variants. Moreover, the selective pressure exerted by CTLs is dependent on the experimental conditions, i.e. the number of added cells and functional activity of the cells. While these parameters are kept as constant as possible between experiments, there is biologic variability that is difficult to control entirely; thus setting RE values based on consensus sequence epitopes provides a frame of reference for comparisons between different experiments and SL9, KF11, and KK10 epitopes, because HIV-1 with consensus sequences in all three epitopes is shared between all libraries. Finally, fitness costs for sequence polymorphisms can vary considerably in different genomic contexts, and our results in HIV-1 strain NL4-3 using single epitope targeting may not reflect the outcome for different virus with CTL pressure on multiple epitopes simultaneously. Related to this point is the inability to assess for compensatory mutations. However, the general patterns we observe are striking, and provide insight into the overall levels of constraints for these epitopes.

In summary, our findings indicate that two immunodominant epitopes associated with protective MHC-I types have highly restricted fitness landscapes for mutation compared to one that is not associated with protection, and that this allows very limited options for escape from CTLs. Additionally, most escape pathways appear to be public and shared between different clones recognizing these epitopes. These results have implications for harnessing CTL responses as vaccines and/or immunotherapies. An early attempt at therapeutic adoptive transfer of CTLs resulted in rapid viral escape [42], and analysis of the failed Step trial demonstrated a “sieve” effect in infected individuals, reflecting viral escape from vaccine-induced CTLs [43]. Thus, a successful CTL-based approach will require understanding of the constraints for escape and strategies to block HIV-1 escape routes through reducing HIV-1 options for mutational escape and/or increasing CTL coverage of mutation options.

## Materials and methods

### Epitope mutational libraries of plasmid HIV-1 NL4-3

Double-stranded DNA spanning the Gag epitope regions of interest were commercially synthesized (gBlock, Integrated DNA Technologies, Coralville, IA) using NNK degenerate codons (where “N” is any nucleotide, and “K” is guanine or thymidine) at each single or double codon position for the epitope and its flanking codons. These gBlock DNA fragments were then PCR amplified using primers 5’-ATCTCTAGCAGTGGCGCCC-3’ with 5’-TTTGGCTGACCTGGCTGTTG-3’ for the fragment containing the SLYNTVATL (Gag 77–85, SL9) epitope, and 5’-AGACACCAAGGAAGCCTTAGATAAGA-3’ with 5’-TACCTCTTGTGAAGCTTGCTCG-3’ for the fragments containing the KAFSPEVIPMF (Gag 162–172, KF11) and KRWIILGLNK (Gag 263–272, KK10) epitopes. These primer sequences corresponded to the start and end sequences of the synthesized DNA fragments. A modified HIV-1 NL4-3 provirus plasmid was created to reduce LTR-driven recombination during cloning, with 5’ U3 and 3’ U5 regions of the HIV LTR removed (to reduce LTR homology), flanked by the CMV immediate-early promoter and the BGH polyA sequence (Fig 1). Additionally, this vector was modified to delete the synthesized epitope regions except the first and last 15 nucleotides; the junction of the deleted regions were modified to have blunt cutting restriction enzyme sites: SfoI for the region containing SL9, AfeI for the region containing KF11 and KK10. After linearizing each plasmid vector with the appropriate enzyme, the PCR-amplified gBlock DNA fragments were inserted via the 15 nucleotide homology by “Infusion” (Clontech, Mountain View, CA) to created whole genome plasmid libraries. The resulting plasmids were then transformed into Stellar chemocompetent *E*. *coli* (Clontech, Mountain View, CA), plated onto 100mm LB/ampicillin plates at ~2x10^4^ colonies/plate and grown for 24 hours at 30°C. Colonies were collected by washing the bacteria from the plates with Luria broth with ampicillin. The plasmid DNA isolated from these bacteria served as the initial “plasmid libraries” for each epitope.

### Creation of HIV-1 epitope libraries

The plasmid libraries of each epitope were lipofected into two T75 flasks of 70% confluent HEK 293T cells (obtained from Dr. Irvin S. Y. Chen, University of California, Los Angeles) using 20μg DNA with BioT lipofection reagent (Bioland Scientific, Paramount, CA). After 24 hours the media was removed, and 10^7^ T1 cells [44] (obtained from Dr. Bruce D. Walker, Harvard University) in 20mL RPMI 1640 medium supplemented with 10% FCS, L-glutamine, HEPES, and penicillin-streptomycin (R10) were added to each flask to promote cell-cell infection of the T1 cells. After 24 hours, the nonadherent cells were removed and transferred to a new flask. These cells were then cultured for 6 to 8 days in R10 media until at least 50% of the cells were infected with HIV-1 (determined by expression of p24 antigen in the cells by intracellular staining and flow cytometry). The supernatant was then filtered through a 0.45 micron filter and cryopreserved to be utilized as the “starting virus library.” All virus libraries were produced in duplicate, and all experiments utilized both libraries in parallel, with duplicates for cultures without CTLs (two replicates for each library, four total) and singles for cultures with CTLs (one replicate for each library, two total).

### HIV-1-permissive cell lines

Cell lines utilized for passaging of HIV-1 included T1[44] (expressing A*02 for the SL9 library and A*02-restricted CTLs), 1CC4.14 cells (expressing B*57 for the KF11 library and B*57-restricted CTLs, previously produced in our laboratory [45]), and Subject 00076 EBV-transformed B-cells (previously produced in our laboratory from PBMC) that were transduced with human CD4 (expressing B*27 for the KK10 library and B*27-restricted CTLs).

### Ethics statement

CTL clones (Table 1) were previously isolated from chronically HIV-1-infected persons and maintained as previously described [46–48] from blood obtained with written informed consent under a University of California, Los Angeles Institutional Review Board-approved protocol, with the exception of 68A62 provided by Dr. Bruce D. Walker (Harvard University).

### HIV-1-specific CTL derivation and maintenance

In brief, peripheral blood mononuclear cells (PBMCs) were enriched for the CTLs of interest by culture with the appropriate epitope, followed by cloning at limiting dilution. Some experiments utilized KK10-specific CTLs previously produced by stable lentiviral transduction of allogeneic CD8^+^ T-cells with a KK10-specific T cell receptor (TCR) sequence identified by quantitative spectratyping [31] (TCR5) that had been cloned into a lentiviral vector as previously described [34]. CTLs were maintained by periodic stimulation with 200ng/mL of the monoclonal anti-CD3 12F6 antibody [49] with irradiated allogeneic PBMCs (obtained anonymously through the UCLA AIDS Institute Virology Core Facility) in R10 media supplemented with recombinant human interleukin-2 (NIH AIDS Reference and Reagent Repository) at 50IU/mL (R10-50). For the CTL clones, TCR beta variable (BV) chain sequences were determined after RNA isolation using Trizol reagent (ThermoFisher Scientific, Waltham, MA), amplification and cloning of the BV gene using the SMARTER 5’ RACE kit (Clontech, Mountain View, CA) with a constant region primer (5’-CTTCTGATGGCTCAAACAC-3’), and sequencing using the same primer.

### Passaging of virus libraries

5x10^6^ permissive cells (10^6^ cells for the SL9 library passaged with the 1.9 CTL) were infected with the starting virus library, yielding about 10–20% infected cells after 72–96 hours (determined by intracellular staining for p24). The cells were then washed twice and resuspended at 5x10^5^ cells/mL in R10-50. CTLs were added at effector:target ratios of 1:8 (except 1:2 for the SL9 library with CTL 1.9), with parallel no-CTL controls. These cultures were fed every 3 days by removing and replacing half of the media. After 7 days the supernatant was filtered through a 0.45 micron filter and cryopreserved; virus in the supernatant was quantified via p24 ELISA (Xpress Bio, Frederick, MD). This virus was utilized to infect cells for a second passage in the same manner using 5x10^3^ pg p24 per 10^6^ target cells (10^3^ pg p24 per 10^6^ target cells for the KK10 library), followed by collection and cryopreservation as before. All passaging with CTLs was performed with duplicate virus libraries, and passaging without CTLs was done in quadruplicate (2 replicates for each virus library).

### Deep sequencing of passaged virus libraries

The passaged virus supernatant was treated with DNAse I (New England Biolabs, Ipswich, MA) to remove residual plasmid DNA. HIV-1 RNA was isolated with the QIAmp viral RNA mini kit (Qiagen, Hilden, Germany), and reverse-transcribed with the high capacity cDNA reverse transcription kit (ThermoFisher Scientific, Waltham, MA) and quantified by real-time PCR with ssoFast EvaGreen supermix on a CFX96 (Bio-Rad, Hercules, CA) with gag-specific primers (5’-ATCTCTAGCAGTGGCGCCC-3’ and 5’-TTTGGCTGACCTGGCTGTTG-3’) compared to NL4-3 plasmid standard to ensure ≥5x10^5^ copies/μL of cDNA per specimen. This cDNA and the starting plasmid libraries were prepared for deep sequencing by PCR amplification using primers tagged with 6 base-pair customized barcodes. The gene specific portions of the primers were:

5’-CCATCCCTTCAGACAGGATCAGA-3’ and 5’-AAGGCTTCCTTGGTGTCTTTTAC-3’ for the SL9 libraries,
5’- AGGCCATATCACCTAGAACTTTA-3’ and 5’- CCCACTGTGTTTAGCATGGTATT-3’ for the KF11 libraries, and
5’-TCCACCTATCCCAGTAGGAGAAA-3’ and 5’-GTCCTTGTCTTATGTCCAGAATGC-3’ for the KK10 libraries.

Deep sequencing was performed with Hiseq PE150 sequencing (Illumina, San Diego, CA).

### Deep sequencing analyses

The sequence data were parsed using the SeqIO function of open source BioPython software (http://biopython.org/). Sequences from different samples were de-multiplexed by the barcodes and mapped to the corresponding region in the HIV-1 genome. Since both forward and reverse reads covered the mutated region, paired reads were used to compensate for sequencing errors. A polymorphism was accepted as valid only if observed in both reads and with a quality score ≥30. Further filtering for errors was done by comparison to control deep sequencing of the index NL4-3 plasmid; variants present at a frequency <10^−4^ were only accepted if their frequencies in duplicate virus libraries exceeded 10-fold the observed frequency of the variant in the control plasmid sequences (due to background error). The sequencing depth was >6x10^5^ and >4x10^6^ for the virus and plasmid libraries respectively. All the data processing and analysis was performed with customized python scripts, which are available upon request. Variants above threshold in initial virus libraries whose frequencies decayed to 0 after passaging were assigned a frequency of 10^−6^ for calculation of RE values. All sequences have been uploaded to GenBank (PRJNA394927).

### HIV-1 clonal mutagenesis of the SL9 epitope

Site-directed mutagenesis was performed with the Q5 mutagenesis kit (New England Biolabs, Ipswich, MA) on the modified pNL4-3 vector described above, which had been further modified to contain the M20A mutation that ablates Nef-mediated MHC-I downregulation [50,51]. The SL9 epitope was modified to create variants SLYNAVAVL (codon 4 = GCT, codon 7 = GTG), SLYNTVACL (codon 8 = TGT), SLYITVATL (codon 4 = ATA), SLYNCVACL (codon 5 = TGT, codon 8 = TGT), SLYCTVATL (codon 4 = TGT), and the resulting plasmids were lipofected into HEK 293T cells as above to produce virus.

### Virus suppression assays

Evaluation of HIV-1 susceptibility to CTL suppression was performed as previously described [32,48]. Briefly, T1 cells [44] were infected with 500pg p24/10^6^ cells of the indicated viruses, and 5x10^4^ infected cells with 5x10^4^ CTL (S2 Fig) or 1.25x10^4^ CTL (Fig 9) were cultured in 200μL R10-50 U/ml IL-2 in a 96 well flat-bottom plate, with monitoring of supernatant p24 antigen by ELISA (Xpress Bio, Frederick, MD).

### Statistics

Comparisons for correlations of replicate experiments and selection of epitope variants by different CTL clones were performed using Spearman rank correlation. Comparisons of means of two groups were performed using Student’s t-test. Comparisons of frequencies between two groups were performed using Fisher’s exact test.

## Supporting information

S1 Fig CTLs utilized in this study suppress HIV-1 replication. T1 cells were infected with HIV-1 NL4-3.1 (with the subtype B consensus SL9 epitope sequence) and co-cultured with CTL clone 1.9, followed by monitoring of supernatant p24 antigen. Results are plotted for viral replication in the presence (closed triangles) or absence (open circles) of CTL co-culture (A). Results for day 6 or 7 are shown for all CTLs utilized in this study (B). Each value is the mean of triplicates, and error bars represent standard deviations.
(TIF)

S2 Fig Schematic describing the passaging of HIV-1 epitope mutant virus libraries under selective pressure from CTLs. Plasmid libraries created as described in Fig 1 were transfected into 293T cells to produce starting virus libraries, which were then passaged in the presence or absence of CTLs for two consecutive rounds of 7 days each. Deep sequencing of the epitope region was performed for the initial plasmid library and the virus libraries before and after selective passaging.
(TIF)

S3 Fig HIV-1 epitope variants with stop codons generally decay during passaging of the epitope variant libraries. For each stop codon present above a threshold frequency of 10^−4^ in the starting virus library, the frequency over time after passaging in the absence of CTLs is plotted. Each value represents the average of experimental duplicates for each library.
(TIF)

S4 Fig Numbers of SL9 epitope variants attaining various thresholds for enrichment by CTLs. For each epitope variant (including immediately flanking residues) with initial library frequencies above 10^−4^ in both replicates, first columns indicate relative enrichment versus consensus without CTLs (RE_-CTL_), second columns indicate relative enrichment with added CTLs (RE_+CTL_), and third columns indicate the difference (ΔRE) as in Fig 4. Fourth, fifth, and sixth columns indicate variants achieving two-fold, five-fold, and ten-fold enrichment (ΔRE ≥ 0.30, 0.70, or 1.0, respectively) by CTLs (open squares) or not (shaded squares).
(TIF)

S5 Fig Numbers of KF11 epitope variants attaining various thresholds for enrichment by CTLs. For each epitope variant (including immediately flanking residues) with initial library frequencies above 10^−4^ in both replicates, first columns indicate relative enrichment versus consensus without CTLs (RE_-CTL_), second columns indicate relative enrichment with added CTLs (RE_+CTL_), and third columns indicate the difference (ΔRE) as in Fig 4. Fourth, fifth, and sixth columns indicate variants achieving two-fold, five-fold, and ten-fold enrichment (ΔRE ≥ 0.30, 0.70, or 1.0, respectively) by CTLs (open squares) or not (shaded squares).
(TIF)

S6 Fig Numbers of KK10 epitope variants attaining various thresholds for enrichment by CTLs. For each epitope variant (including immediately flanking residues) with initial library frequencies above 10^−4^ in both replicates, first columns indicate relative enrichment versus consensus without CTLs (RE_-CTL_), second columns indicate relative enrichment with added CTLs (RE_+CTL_), and third columns indicate the difference (ΔRE) as in Fig 4. Fourth, fifth, and sixth columns indicate variants achieving two-fold, five-fold, and ten-fold enrichment (ΔRE ≥ 0.30, 0.70, or 1.0, respectively) by CTLs (open squares) or not (shaded squares).
(TIF)
