# Supplementary figures and images for: HIV-1 epitopes presented by MHC class I types associated with superior immune containment of viremia have highly constrained fitness landscapes
Source: PLoS Pathog. 2017 Aug 7;13(8):e1006541. doi: 10.1371/journal.ppat.1006541 (PMC5560751; doi:10.1371/journal.ppat.1006541)

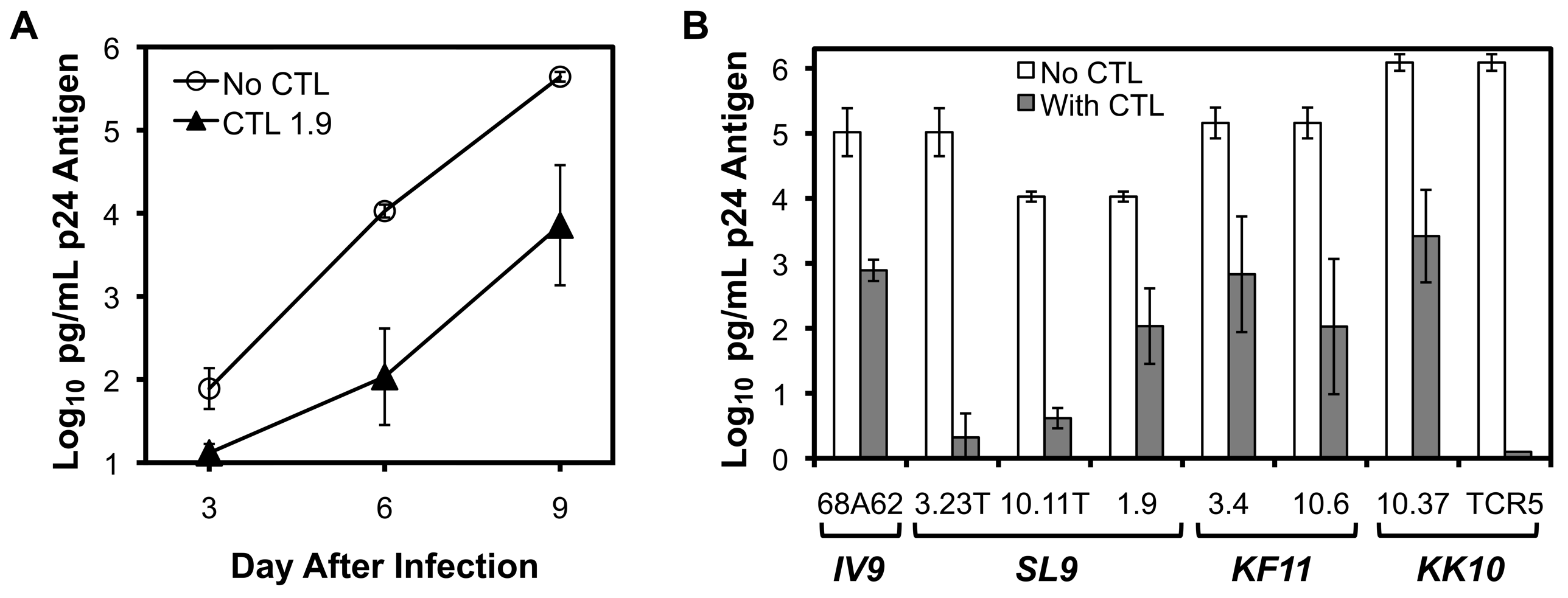

Supplement: S1 Fig — T1 cells were infected with HIV-1 NL4-3.1 (with the subtype B consensus SL9 epitope sequence) and co-cultured with CTL clone 1.9, followed by monitoring of supernatant p24 antigen. Results are plotted for viral replication in the presence (closed triangles) or absence (open circles) of CTL co-culture (A). Results for day 6 or 7 are shown for all CTLs utilized in this study (B). Each value is the mean of triplicates, and error bars represent standard deviations. (TIF) [file ppat.1006541.s001.tif]

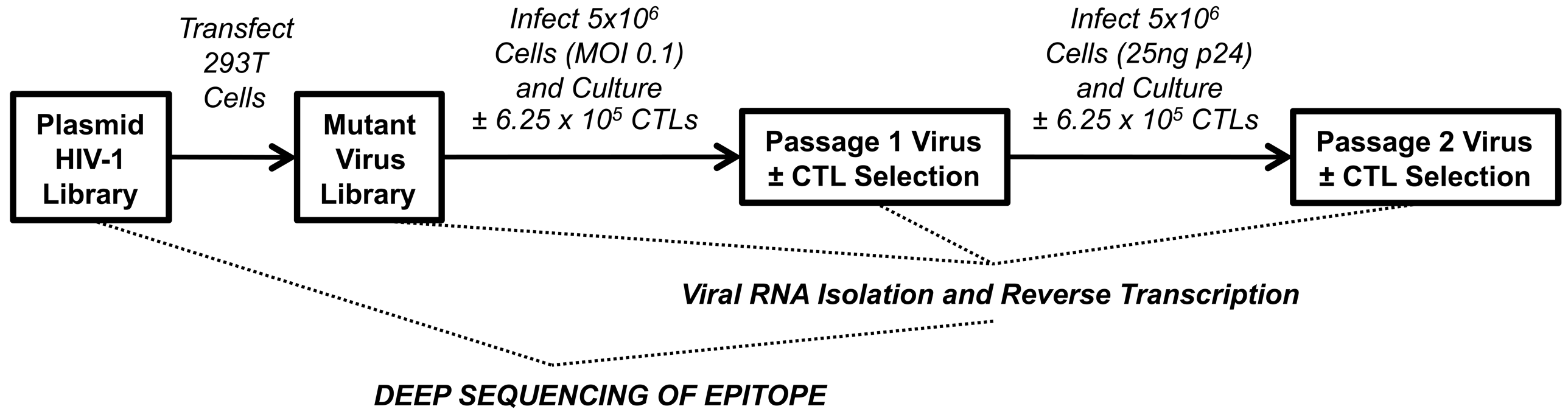

Supplement: S2 Fig — Plasmid libraries created as described in Fig 1 were transfected into 293T cells to produce starting virus libraries, which were then passaged in the presence or absence of CTLs for two consecutive rounds of 7 days each. Deep sequencing of the epitope region was performed for the initial plasmid library and the virus libraries before and after selective passaging. (TIF) [file ppat.1006541.s002.tif]

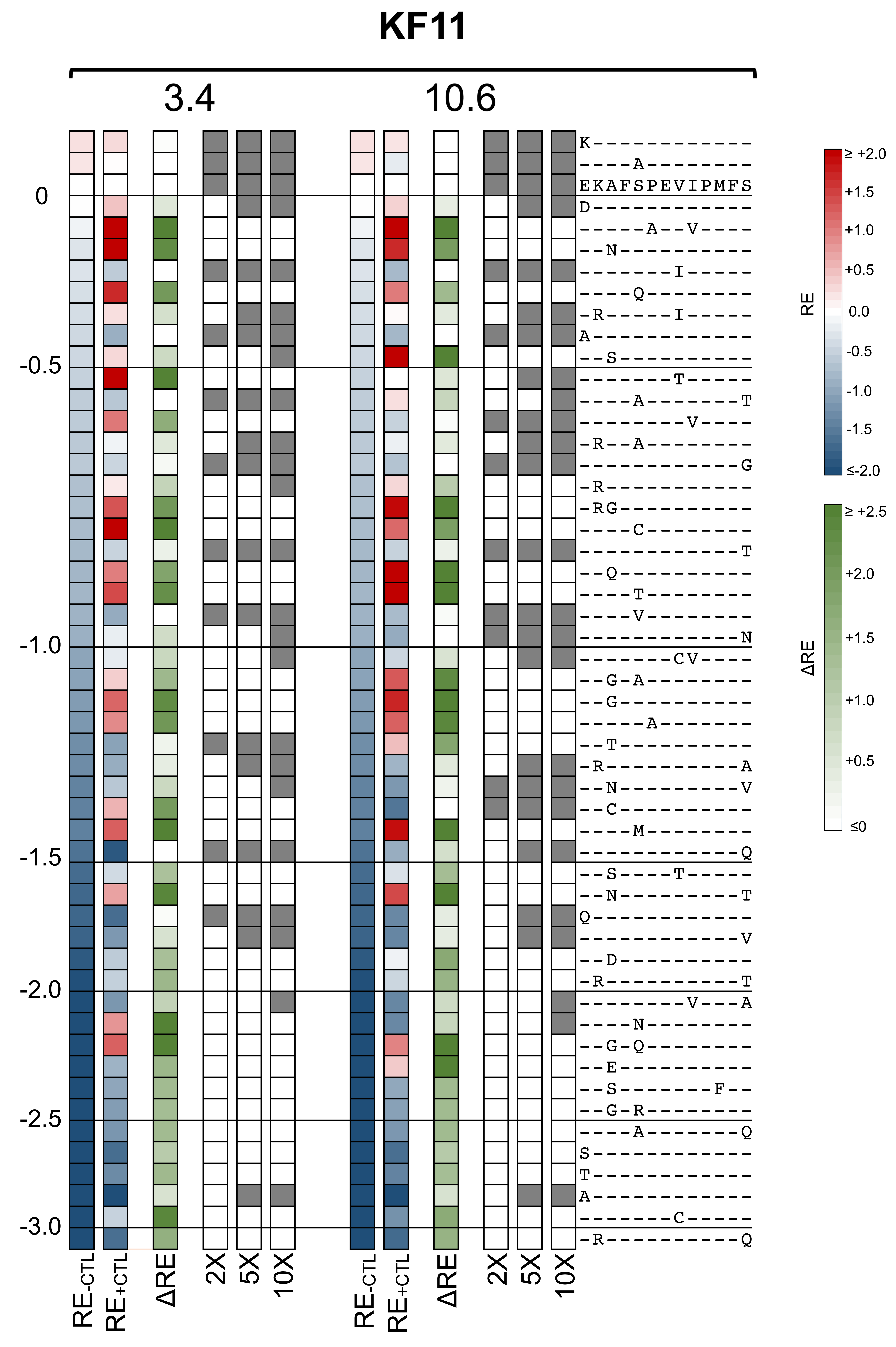

Supplement: S5 Fig — For each epitope variant (including immediately flanking residues) with initial library frequencies above 10−4 in both replicates, first columns indicate relative enrichment versus consensus without CTLs (RE-CTL), second columns indicate relative enrichment with added CTLs (RE+CTL), and third columns indicate the difference (ΔRE) as in Fig 4. Fourth, fifth, and sixth columns indicate variants achieving two-fold, five-fold, and ten-fold enrichment (ΔRE ≥ 0.30, 0.70, or 1.0, respectively) by CTLs (open squares) or not (shaded squares). (TIF) [file ppat.1006541.s005.tif]
